# Current curricular trends after the COVID-19 pandemic: A national survey of psychiatry residency programs

**DOI:** 10.1177/00912174231152575

**Published:** 2023-01-18

**Authors:** Jeff Wang Jin, Kacy Smith, Amanda Helminiak, Vineeth John, Hanjing Emily Wu

**Affiliations:** 1 12339 The University of Texas Health Science Center Houston – McGovern Medical School, Houston, TX, USA; 2 Department of Psychiatry and Behavioral Sciences, 12339 The University of Texas Health Science Center Houston – McGovern Medical School, Houston, TX, USA; 3 144383 The University of Texas Health Science Center Houston – Harris County Psychiatric Center, Houston, TX, USA

**Keywords:** psychiatry, medical education, curriculum development, COVID-19, residency

## Abstract

**Objectives:**

During the COVID-19 pandemic, many psychiatry residencies (academic, community, and hybrid programs) have adopted different learning modalities to preserve a high quality of educational training. There is minimal data on specific program adaptations, related change perspectives, and program type stratification. This study sought to examine trends in curriculum changes in accredited psychiatry residency programs in the United States.

**Methods:**

Program directors of accredited general psychiatry programs in the United States were surveyed to assess both general program details and changes in educational content, delivery, and perspectives with regard to program curricula.

**Results:**

A total of 63 program directors out of 264 eligible programs completed the questionnaire (23.9%). There was a significant shift to integrate virtual learning post-pandemic (98.5%) compared to pre-pandemic (3%). However, there was no association between these modality changes and program type (*p* = 0.13). Furthermore, changes were noted with respect to didactic content (60%), decreased rotation sites (38%), and increased telemedicine exposure (84%) with no change of wellness days (67%) or protected time (97%). Again, the above changes had no association with program type. Use of virtual educational platforms was described as positive (59.7%) with 9 programs noting a future transition to a hybrid learning model for didactics and grand rounds.

**Conclusions:**

The findings suggest that pandemic-related curriculum adaptations occurred in all different types of psychiatry residencies and suggest a national trend to continue virtual educational platforms with regard to psychiatry didactics. However, future investigation of effectiveness of virtual learning programs in psychiatry residencies is warranted.

## Introduction

Psychiatry residency education has evolved in response to social, economic, and cultural forces that shape the patterns of practice. Over the past 75 years, psychiatry education has shifted from irregularly structured curricula to more regulated process and requirement-driven models.^1^ Today, residents train in a system that has adapted to promote competency through clinical, didactic, and evidence-based learning. These include direct patient management, grand rounds, didactic education, and journal clubs. Historically, trainees have had the option to train at either community-based or university-affiliated academic hospitals, each with unique advantages. Community hospitals are generally smaller, more closely aligned with the community, and provide practical experience in settings residents are likely to practice in upon graduation.^2,3^ In comparison, academic hospitals are generally noted for increased patient diversity, resource-intensive services, and opportunities for post-residency training.^4^ However, the common mission to advance medical education and patient care has resulted in the production of hybrid models that have characteristics of both academic and community programs.^5^

In March 2020, the COVID-19 pandemic completely shifted the landscape of residency education. In response to the lockdown, several tactics were used to maintain the delivery of quality education while mitigating the risks of the potential infection from the virus. Education shifted from the classroom to virtual spaces, clinical learning was replaced with remote modular learning, and services were reconfigured to ensure the mental health and well-being of residents.^6^ Some residency programs substituted simulations for procedures and used team rotation systems to optimize the number of in-hospital residents for safety reasons.^7^ Non-immune to the pandemic, psychiatry programs may have needed to adopt many of these adaptations to maintain a quality educational experience for residents. Furthermore, the pandemic also negatively affected the mental health and quality of life for residents.^8^ Psychological pressure with managing COVID-19 positive patients with limited protective equipment, isolation from family in fear of infection transmission, and excessive working hours may have contributed to burnout and unhealthy mental states that impair learning.

To date, there are minimal studies that have examined the adaptations made specifically for psychiatry residency programs secondary to the pandemic. The perception of these changes amongst educational psychiatry leaders also remains uncharted. Additionally, no studies have investigated the pandemic-induced changes stratified based on program type. It remains unclear whether certain adaptations demonstrated more benefit in a particular program type. This study aims to analyze curricular trends across accredited psychiatry residency programs in the United States to gain knowledge on how to continue the innovation of psychiatry education.

## Methods

### Survey Design

The constructed survey aimed to quantify pandemic-induced psychiatry residency curriculum changes and assess psychiatry faculty perceptions towards the evolving educational landscape. Composed of 15 total items (12 multiple choice, 3 free response), the survey was developed into three sections: demographics, program changes, and director perceptions. The full survey form can be seen below (Table 1).

**Table 1.** Abbreviated study survey.

Primary program categorization
Academic
Community
Hybrid
Majority format of pre-pandemic education
Virtual
In-person
Mixed
Majority format of post-pandemic education
Virtual
In-person
Mixed
Didactic changes were made in response to the pandemic
True
False
If true, please describe:
Free Response
Changes to rotation/clinical elective sites in response to the pandemic
Increased
Decreased
Unchanged
If true, please describe:
Free Response
Wellness days/didactics are included in the residency curriculum
True
False
If true, how has wellness time changed in response to the pandemic
Increased
Decreased
Unchanged
Changes to resident protected time in response to the pandemic
Increased
Decreased
Unchanged
Telemedicine exposure is included in the residency curriculum
True
False
If true, how has telemedicine changed in response to the pandemic
Increased
Decreased
Unchanged
Perception of curricular changes in response to the pandemic
Positive
Negative
Neutral
Perception of integration of virtual learning in response to the pandemic
Positive
Negative
Neutral
Future curricular considerations in response to the pandemic
Free Response

First, respondents provided information regarding program institution type (academic, community, hybrid) and resident learning modalities (pre- and post-pandemic). Next, respondents were surveyed on curricular changes in didactic content, clinical rotation sites, resident wellness, and telemedicine exposure secondary to the pandemic. Finally, respondents were assessed on their attitudes towards respective pandemic changes and considerations for future curriculum adaptations.

### Study Recruitment

The study protocol was approved by the appropriate Institutional Review Board. A total of 277 psychiatry residency program director emails were manually located through the Electronic Residency Application Service (ERAS) and public contact information on respective program websites. Although ERAS primarily serves as a residency application platform, this study used it as a source to obtain the most updated contact information for psychiatry residency program directors. Any program director emails that were not listed on the ERAS database were further pursued through public internet Web site of each psychiatry residency program. Surveys were sent to the listed contact emails for psychiatry residency programs between November 2021 and December 2021. Of these, 13 emails (4.7%) were returned as undeliverable without any alternative method of contact. Of the remaining 264 eligible emails, 63 survey responses (23.9%) were obtained anonymously through Qualtrics software. Although the direct emails had clear instructions for program directors use, there was no official verification process to ensure that the actual program director of the psychiatric residency program the sole respondent to the survey.

### Data Extraction

All quantitative data for statistical analysis was extracted from the Survey directly. Qualitative data was obtained as a format of freestyle elaboration.

### Statistical Analysis

Due to limited sample sizes, Fisher’s Exact test was used to examine differences between program types (academic, community, and hybrid) across several variables (changes in content, delivery modality, resident wellness, telemedicine exposure, and director perspectives). Chi-squared analysis was also performed similarly when an appropriate sample size was present. All analyses were conducted with a statistical significance level at *P* < .05 through SPSS 26 software.

## Results

### Demographics

Of 264 eligible programs, 63 program directors responded. Among these responses, the self-identified distribution showed 31 academic (49.2%), 19 community (30.2%), and 13 hybrid (20.6%) programs (Table 2). Due to the anonymity of the survey, the exact geographical details of the respondents remain unknown.

**Table 2.** Psychiatry residency curricular trends after the COVID-19 pandemic.

					Statistical Analysis^a^	
	Total, *N* (%)	Academic, *N* (%)	Community, *N* (%)	Hybrid, *N* (%)	Fisher’s Exact^b^	Chi-Square (χ2)
Pre-pandemic Format						
In person	61 (97)	31 (100)	17 (90)	13 (100)	*P* = .2539	
Virtual	1 (1.5)	0 (0)	1 (5)	0 (0)		
Mixed	1 (1.5)	0 (0)	1 (5)	0 (0)		
Post-pandemic Format						
In person	1 (1.5)	0 (0)	0 (0)	1 (7.7)	*P* = .1343	
Virtual	33 (52.5)	13 (42)	12 (63)	8 (61.5)		
Mixed	29 (46)	18 (58)	7 (37)	4 (30.7)		
Didactic Content Changes						
Yes	38 (60)	16 (52)	11 (58)	11 (85)		χ2 = 4.23
No	25 (40)	15 (48)	8 (42)	2 (15)		*P* = .1206
Rotation and Elective Sites						
Unchanged	39 (62)	20 (65)	12 (63)	7 (54)		χ2 = 0.46
Decreased	24 (38)	11 (35)	7 (37)	6 (46)		*P* = .7945
Increased	0 (0)	0 (0)	0 (0)	0 (0)		
Wellness Days						
Unchanged	42 (67)	22 (71)	12 (63)	8 (61)	*P* = .7129	
Decreased	2 (3)	1 (3)	0 (0)	1 (8)		
Increased	19 (30)	8 (26)	7 (37)	4 (31)		
Protected Time						
Unchanged	61 (97)	29 (94)	19 (100)	13 (100)	*P* = .6984	
Decreased	0 (0)	0 (0)	0 (0)	0 (0)		
Increased	2 (3)	2 (6)	0 (0)	0 (0)		
Has Current Telemedicine Exposure						
Yes	52 (83)	26 (84)	14 (74)	12 (92)	*P* = .4173	
No	11 (17)	5 (16)	5 (26)	1 (8)		
Telemedicine Exposure post-pandemic						
Unchanged	10 (16)	5 (16)	4 (21)	1 (8)	*P* = .6074	
Decreased	0 (0)	0 (0)	0 (0)	0 (0)		
Increased	53 (84)	26 (84)	15 (79)	12 (92)		
General Perspective on Individual Program Changes						
Positive	27 (45.8)	11 (36.7)	11 (64.7)	5 (41.7)	*P* = .3371	
Negative	11 (18.6)	8 (26.6)	1 (5.9)	2 (16.6)		
Neutral	21 (35.6)	11 (36.7)	5 (29.4)	5 (41.7)		
Perspective on Use of Virtual Modalities						
Positive	37 (59.7)	14 (46.7)	12 (66.7)	9 (75)	*P* = .1038	
Negative	10 (16.1)	9 (30)	1 (5.6)	0 (0)		
Neutral	15 (24.2)	7 (23.3)	5 (27.7)	3 (25)		

^a^Significance level was set at *P* < .05.

^b^Fisher's Exact Test was performed in substitution for the Chi-Square Test for small sample categories (*n* < 2).

### Learning Modality and Content

For content delivery, the majority of respondents reported primary use of in-person modalities before the pandemic (97%). Few program directors reported using virtual (1.5%) or mixed (1.5%) modalities pre-pandemic. Post-pandemic, respondents reported primary use of virtual (52.5%) and mixed (46%) modalities to facilitate resident learning. One program directors reported continued use of an in-person format after the onset of the pandemic. When compared across program types, pre-pandemic [*P* = .2539] and post-pandemic [*P* = .1343] content delivery modalities showed no significant differences between academic, community, and hybrid psychiatry residencies. Furthermore, the majority of program directors reported making changes to their resident didactic content (60%) in response to the pandemic. However, there was no significant difference in didactic content changes across academic, community, and hybrid programs [*P* = .1206, *χ2* = 4.23].

### Resident Wellness

In terms of resident wellness days, most respondents reported no changes in the number of wellness days (67%) and allotted protected time (97%). Moreover, some programs respondents indicated an increase in the number of resident wellness days (30%). In contrast, a few program directors reported decreased wellness days (3%) and increased protected time (3%). When compared across program types, resident wellness days [*P* = .7129] and protected time [*P* = .6984] showed no significant differences between academic, community, and hybrid psychiatry residencies.

### Rotation Sites and Telemedicine

When surveyed about total rotation and elective sites, respondents reported an unchanged (62%) or decreased (38%) number secondary to the pandemic. No program directors reported increases in rotation sites. Additionally, most program directors reported the use of current telemedicine exposure (83%) with increased exposure post-pandemic (84%). Noteworthy, very few program directors reported lack of telemedicine use (17%) and unchanged exposure secondary to the pandemic (16%). No program directors reported decreases in telemedicine exposure after the pandemic. When compared across program types, there was no significant difference regarding changes in rotation sites [*P* = .7945, *χ2* = 0.46], current telemedicine use [*P* = .4173], and post-pandemic telemedicine exposure [*P* = .6074] between academic, community, and hybrid programs. This is the first study conducted to compare the curricular changes between academic, community and hybrid General Psychiatry residency programs post COVID-19 pandemic. Although no significant curriculum changes were found among above programs, it indicates a general trend of residency curriculum among different types of residency programs in future years.

### Perspectives and Future Changes

When asked about the perspectives on individual program changes, most respondents reported positive (45.8%) or neutral (35.6%) attitudes towards respective changes. Surprisingly, few program directors reported negative perceptions (18.6%) on their individual respective changes. Furthermore, respondents reported positive attitudes (59.7%) towards the integration of virtual learning modalities into psychiatry residency training, in contrast only 24.2% of program directors reported neutral attitude and 16.1% of program directors reported negative attitudes towards the use of virtual learning platforms. When compared across program types, there was no significant difference regarding perspectives on individual program changes [*P* = .3371] and virtual learning integration [*P* = .1038] between academic, community, and hybrid programs.

Respondents were also given the opportunity to elaborate on the considerations for future program changes through free response. We found the most common qualitative themes included usage of a hybrid learning model (virtual/in-person) for didactic-based learning (*n* = 9), willingness to return to in-person activities when deemed safe (*n* = 5), increased telemedicine use and didactics (*n* = 2), and increased didactics on disaster management and contingency planning (*n* = 1).

## Discussion

### Educational Innovation

Although insignificant, our results demonstrated three major shifts in the educational landscape of psychiatry residency education: adoption of virtual learning, adaptation of didactic content, and increased use of telemedicine. These were integrated as an emergent response to prevent the potential development of educational deficits secondary to the pandemic. While these shifts are not unique to only psychiatry residencies, they highlight an intriguing avenue for further curriculum innovation.

The review by Kaul et al. advocates for the digitalization of didactic sessions, grand rounds, and journal clubs in resident education.^9^ Alternative team management, game-based learning, and social media platforms may be used in conjunction for repetition and reinforcement. However, it is also imperative to emphasize non-clinical learning to prevent economic repercussions and promote diversity, equity, and inclusion (DEI). Investments in financial literacy, resources for implicit bias education, and the mitigation of potential stressors (child or elder care) can improve the learning experience of trainees. For psychiatry education, the narrative review from Ho et al. also supported the integration of technology-enhanced learning modalities (remote teaching, virtual conferences, telemedicine).^10^ However, our results suggest that the pandemic decreased clinical rotation sites for residents. Some programs have also shifted more focus from traditional didactic topics to more recent pandemic-related topics. These factors may result in small but exigent deficits in clinical and didactic exposure.

Online curricula may be quality supplements to traditional psychiatry residency training. The American Association for Geriatric Psychiatry (AAGP) created a web-based curriculum designed as introductory exposure to geriatric psychiatry for residents and trainees.^11^ Field experts recorded 30 lectures focused on the assessment, diagnosis, and treatment of older adults aimed to address pandemic-induced learning gaps during in-person clinical experiences. The National Neuroscience Curriculum Initiative (NNCI) is another supplement to neuroscience education in psychiatry trainees.^12^ While it intended to focus on improving the dissemination of educational neuroscience resources, the curriculum has been implemented in over 200 psychiatry residency programs — even through the pandemic. When properly refined, online curricula may be an innovative way to supplement psychiatry learning where clinical experience is negatively affected.

Similar to the residency match, the pandemic may have restricted opportunities for psychiatry residents to travel and collaborate with colleagues from other institutions.^13^ The creation of consortia may provide cross-institutional opportunities for education and networking. The Otolaryngology Program Directors Organization (OPDO) created 3 consortia that offered live and recorded lectures over web-based teleconferencing by faculty from numerous national institutions. Lecture times were staggered for learning convenience in various time zones and supplemental materials were provided for structure. These tactics aimed to combat the reduced clinical experience, educational resources, and in-person contact secondary to the pandemic. However, Xie et al. noted significant obstacles of uncompensated time, expenses, management, and maintenance with the founding of these consortia.^13^ Consortia may improve psychiatry education, but it is imperative to consider sustainability. Local consortia composed of neighboring or regional institutions may be an intriguing concept that can provide enhanced resident learning, cross-institutional networking, and cheaper sustainability.

### Resident Wellness

Mental health repercussions from the pandemic are on the rise. In primary and surgical residents, the pandemic has affected social interactions, spiritual well-being, and increased levels of mental and physical exhaustion.^14^ Hence, residency programs have increasingly prioritized resident wellness to mitigate further deterioration of mental health.^15^ Generally, residents are allocated protected time and wellness days to address both personal and professional needs. However, our results showed that most programs did not change the number of wellness days and protected time in response to the pandemic. Only a subset of nearly one-third of program directions indicated an increase in the total number of wellness days. One review identified that resident well-being was associated with personal time availability (PTA), the reported ability of residents to find time to exercise, socialize, and tend to errands.^16^ Residents with higher PTA scores reported more positive experiences/emotions, increased career choice satisfaction, and less perceived stress. Given the impact of the pandemic on resident wellness, it is imperative to use evidence-based strategies to adapt to residents’ well-being needs.

During the pandemic, one surgical residency program aimed to optimize residency wellness by using a model that emphasized a culture of wellness, personal resilience, and efficiency of practice.^17^ To develop wellness culture, the model prioritized open educational discussion of burnout symptoms and even classified burnout as an illness. Residents with any symptoms of illness, including burnout, were mandated to stay at home. In this model, residents reported increased feelings of support if they were physically or mentally unwell. Patient interactions were minimized to promote resident safety and practice efficiency. COVID-19 suspected or positive patients were only examined by either a senior resident or attending. While exposure to COVID-19 patients has been associated with increased physician trainee stress and burnout, it is also important to find an exposure balance that both promotes resident wellness and delivers high-quality clinical training.^18^

### Director Perspectives

While the pandemic heavily influenced the use of virtual learning, psychiatry residency program directors from our study heavily favored the integration of remote education into existing curricula, as concluded from the qualitative data from the survey. Our results show the presence of these shared perspectives on virtual learning across all program types. Despite our reliance on its adoption, virtual learning has several important weaknesses when compared to traditional education methods. One systematic review noted the issues of technical challenges, confidentiality issues, reduced student engagement, and loss of assessments.^19^ These challenges may contribute to decreased motivation, concentration, and learning effectiveness in virtual trainees. Additionally, these factors may be difficult to assess through remote learning and remain without intervention. For prevention, Ho et al. recommended the incorporation of adequate breaks, wellness check-ins, peer support, and live video feeds.^10^ These tactics may encourage trainee engagement, facilitate feedback communication, and improve the education quality trainees receive.

Due to the limitations of virtual learning, several psychiatry program directors reported considering the use of hybrid learning models — a blend of virtual and in-person learning. Where applicable, this combination allows for the achievement of a more optimal balance between the benefits and drawbacks of remote learning. Some non-psychiatry residency and fellowship programs have noted success with the model and report it as a method to adjust and maximize trainee learning.^20,21^ While in-person training may be more traditional, future considerations involving technological innovation and didactics on disaster management may allow psychiatry trainees to become better equipped in the case of another similar crisis.

### Limitations of Virtual Learning

The COVID-19 pandemic has induced the integration and increasing reliance on virtual platforms in residency education. However, concerns remain regarding the consequences of the pandemic interference of psychiatric training potential effects on clinical competency. There is limited current data that demonstrates the long-term outcomes of virtual education in psychiatry residency training. Based on the current available data, the literature shows dramatic interruptions in clinical medical student education in various countries including the United States.^22,23^ These students expressed disruptions due to cancelled in-person clinical instruction, willingness to accept the risks of COVID-19 infection to return to clinical rotations, and even considered repeating the final year of training to supplement decreased clinical experience.^22,23^ Specifically for psychiatry training, medical students in the United Kingdom experienced reduced educational demands such as experience documentations, attendance monitoring, workplace-based assessments, and case presentations, but noted no lowering or compromised of clinical standards.^24^ Final year medical students in Sri Lanka also reported great impacts on psychiatric training and exam performance. The pandemic had impacts on clinical aspects including patient availability, mental status assessments, rapport development, diagnostic skills, and clinical exam components.^25^ Although there is limited data specifically involving psychiatry residency training in the United States, psychiatry residency shares some similar clinical aspects of medical training. The pandemic impact on clinical involvement in medical student training suggests the possibility of interruptions in clinical residency training and risks of clinical competency despite further supplementation with virtual and hybrid learning modalities.

### Study Limitations

Our results should be interpreted in the context of some limitations. While appropriate for statistical power, the response rate we received was lower than expected. Due to anonymous response designed in the study, we cannot inquire those non-responded programs to respond. The small response rate (23.9%) limits the generalizability of our results to psychiatry programs throughout the United States. Additionally, the majority of responses to the open-ended question in the survey remained unanswered, opening the possibility of non-response bias. Initially, free response questions were limited to potentially encourage increased survey completion. However, knowledge of the reasoning behind responses regarding perspectives would have also been useful. Thus, respondents should have also been given the opportunity to justify the reasoning for their perspective selections to further inform future academic progress. Furthermore, the study evaluated qualitative changes and perspectives, but did not include measures on the effectiveness of these changes. This may suggest a limited scope with need for further investigation.

## Conclusion

The COVID-19 pandemic forced psychiatry residency programs to innovate education delivery. Our findings suggest that these innovations were ubiquitous, regardless of program type. Although difficult to navigate, the pandemic has highlighted several adaptations that may have the considerate potential to further innovate psychiatry education. Hybrid learning has remained a popular post-pandemic alternative that appears to streamline learning efficiency and promote trainee wellness. As changes continue to be made, it is important to use trainee feedback to improve chances for long-term sustainability. Regardless, psychiatry education must continue to seize the momentum of educational improvement created by the pandemic. Further investigation is needed on the effectiveness and long-term outcomes of these learning adaptations to ensure efficacy.
